# Biased Signaling Agonists of Dopamine D3 Receptor Differentially Regulate the Effects of Cocaine On Dopamine Transporter Function

**DOI:** 10.1021/acschemneuro.5c00076

**Published:** 2025-06-26

**Authors:** Sophie R. Cohen, Wei Xu, Nastaran F. Aziz, Rodrigo A. España, Sandhya Kortagere

**Affiliations:** 1 Dept. of Microbiology & Immunology, 12312 Drexel University College of Medicine, Philadelphia, Pennsylvania 19129, United States; 2 Dept. of Neurobiology & Anatomy, 12312 Drexel University College of Medicine, Philadelphia, Pennsylvania 19129, United States

**Keywords:** beta-arrestin2, cocaine self-administration, dopamine D3 receptor, dopamine transporter, fast scan cyclic voltammetry, G-protein biased agonist, phosphorylation, pramipexole, SK609, cocaine seeking

## Abstract

Cocaine use disorder is a major healthcare issue with no effective FDA-approved treatments. Cocaine exerts its effects – in part – by blocking dopamine transporters (DAT) and subsequently dysregulating DAT function. Several molecular targets have been identified as key regulators of DAT function and expression including dopamine D3 receptors (D3R) that are highly expressed in the mesolimbic dopamine pathway. Although D3R partial agonists and antagonists have been shown to influence cocaine seeking in rodents, effects have been inconsistent with studies reporting varying outcomes on cocaine-associated behavior. In this study, we tested the effects of SK609, a novel G-protein biased D3R agonist, and pramipexole, an unbiased agonist of D3R, on DAT expression and function and cocaine-seeking behavior. Results indicated that SK609 reduced phosphorylation of DATs following cocaine and the uptake inhibition effects of cocaine on dopamine transmission in *in vitro* and *ex vivo* studies, respectively. By comparison, pramipexole augmented the effects of cocaine on DAT phosphorylation, enhanced dopamine levels, and increased cocaine seeking in rats. These results suggest that unbiased D3R activation promotes the effects of cocaine and that limiting D3R agonists to G-protein signaling pathways may have the potential to reduce these effects.

## Graphical abstract

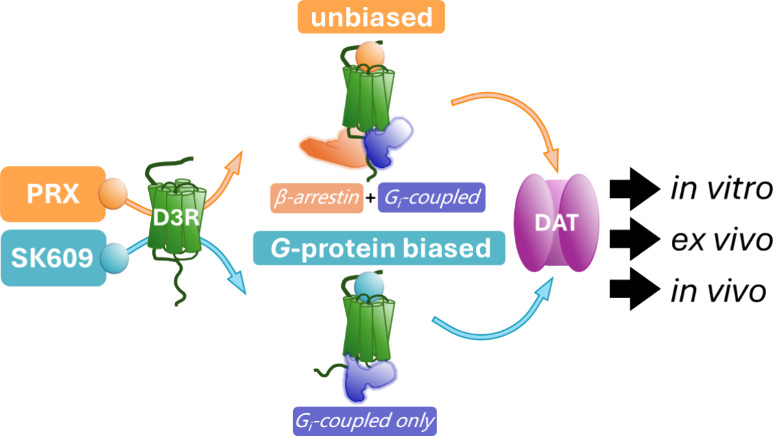

## Introduction

It is well accepted that the mesolimbic dopamine (DA) system is critically involved in the reinforcing effects of cocaine and other drugs of abuse. Nonetheless, the processes by which exposure to cocaine promotes adaptations in mesolimbic DA systems, including the DA transporter (DAT), remain unclear despite decades of research.^1–7^ Cocaine exerts some of its psychoactive effects by inhibiting DATs, leading to an accumulation of DA in the extracellular space. Notably, phosphorylation of the DAT at the Threonine 53 (Thr53) residue (pDAT) is known to sensitize the DAT to the effects of cocaine (i.e., increase inhibition of DA uptake), thereby promoting greater DA levels.^8–10^ Increases in pDAT in the nucleus accumbens (NAc) core have been associated with increases in cocaine self-administration^10^ and cue-induced cocaine seeking during abstinence.^11^ Further, cocaine alters transcription of genes encoding G-protein subunits (e.g., Gβ, Gγ)^12^ and promotes activation of G-protein coupled receptors resulting in increased phosphorylation and subsequent activation of extracellular signal-regulated kinase 1/2 (ERK1/2)^13,14^ and protein kinase C (PKC).^15^ One such example is the dopamine D3 receptor (D3R), especially considering their high sensitivity to DA^16,17^ and high density of expression within the striatum,^18–20^ which make these receptors particularly vulnerable to the effects of cocaine (i.e., DA accumulation caused by DAT blockade).

D3Rs belong to the class of inhibitory DA D2-like receptors that couple to Gi/o type G-proteins and are expressed in the islands of Calleja, NAc, septum, olfactory tubercle, ventral pallidum, substantia nigra pars compacta, dentate gyrus of hippocampus, and prefrontal cortex.^18–20^ Notably, the distribution of D3R within the NAc varies between the core and shell regions, with denser expression of D3R in the NAc shell than in the core.^20–22^ This unique distribution of D3Rs is known to govern a variety of DA-dependent behaviors, including learning and motivation in psychostimulant and alcohol use disorders,^23–28^ cue- and stress-induced drug seeking^6,23,29–31^ and compulsive and impulsive behaviors.^17,32–34^ Others studies demonstrate that D3R agonists, partial agonists, and antagonists, including quinpirole, BP-987, and SB-277011-A, respectively, dose-dependently reduce cocaine seeking.^24,35–39^ Similarly, D3Rs are recruited following cocaine exposure,^40–43^ with increases in D3R expression in the NAc observed in both cocaine-experienced rodents and cocaine users.^18,38,44–49^

Among the many factors that influence DAT function, the D3R stands out as a potential critical participant in the regulation of DAT expression and function. However, the effects of D3R agonists and antagonists on DAT remain unclear with some studies reporting increases,^37,47,50–55^ decreases,^36,37,56^ or no effects^50^ on cocaine-associated behavior. A major limitation in our understanding of D3R regulation of DAT function is the lack of pharmacological tools that selectively target D3R and the potential effects of the D3R signaling pathways. To overcome these limitations, we developed SK609, a selective and G-protein biased D3R agonist with minimal β-arrestin2 recruitment resulting in rapid internalization of D3Rs and no long-term desensitization.^57–59^ Recently, we demonstrated that SK609 and its analogs promote short-term activation of ERK1/2, which is predominantly mediated by G-protein signaling, while the unbiased agonist pramipexole (PRX) promotes both short- and long-term activation of ERK1/2 mediated by β-arrestin2 recruitment.^59^ Since ERK1/2 is known to phosphorylate DAT at Thr53,^8,9^ we anticipate that biased and unbiased signaling agonists of D3R may promote differential phosphorylation patterns of DAT that may influence DAT function and behavioral responses to cocaine.

In the present studies, we hypothesized that biased agonism of D3R with SK609 would decrease DAT phosphorylation and thereby mitigate neurochemical and behavioral responses to cocaine, while PRX would augment cocaine-sensitized neurochemical changes and behavior due to the unbiased recruitment of β-arrestin2. To test this hypothesis, we employed Western blotting to measure the expression of phosphorylated and total DAT levels from cells treated with SK609 and PRX with and without pretreatment with cocaine. Further, we examined DA transmission, as a measure of DAT efficiency and sensitivity to cocaine using *ex vivo* fast scan cyclic voltammetry (FSCV), and finally, we investigated how these changes in DAT function contributed to behavioral effects through cue-induced cocaine seeking during abstinence.

## Results and Discussion

Given the poorly characterized interactions between D3R and DAT proteins, our experiments explored the differential effects of biased signaling cascades from D3R activation on DAT expression, phosphorylation, function, sensitivity to cocaine, and subsequent cocaine seeking behavior. Previous studies demonstrated that D3R activation promotes physical interactions between D3R and DAT.^41,60–63^ Therefore, we explored the potential effects of these interactions within the NAc core because it exhibits dense expression of both D3R and DAT and has been shown to mediate cocaine seeking behavior via D3R manipulation.^64^

### PRX and SK609 Have Opposing Effects on pDAT Expression in Naive and Cocaine-Treated Cells

To examine the differential signaling effects of D3R activation, we utilized our previously developed human SH-SY5Y neuroblastoma cell line with stable expression of D3R (SH-SY5Y-D3R).^59^ In this study, we tested the temporal effects of PRX (5 nM) or SK609 (1 μM) alone (Figure 1A–D) as well as the effects of saline, PRX, or SK609 following cocaine (10 mM) exposure (Figure 1E–H) on total DAT (tDAT) and pDAT expression in SH-SY5Y-D3R cells using Western blotting. Concentrations of PRX and SK609 (10xEC50 value at D3R) were chosen based on our previous studies.^59^ Neither PRX (*n* = 3) nor SK609 (*n* = 3) treatment influenced expression of tDAT (Figure 1A,B). A two-way repeated measures ANOVA revealed no main effect of treatment [F­(1, 32) = 0.4275, *p* = 0.518], time [F­(7, 32) = 0.1043, *p* = 0.998], or of a treatment × time interaction [F­(7, 62) = 0.04957, *p* > 0.999] on tDAT expression. Notably, PRX produced peak pDAT expression to a greater extent and for a longer duration than SK609 (Figure 1C). A two-way repeated measures ANOVA revealed a significant effect of treatment [F­(1, 32) = 402.7, *p* < 0.001], time [F­(7, 32) = 57.18, *p* < 0.001], and a treatment × time interaction [F­(7, 32) = 28.35, *p* < 0.001] on pDAT expression. Šidák posthoc tests revealed significant differences between PRX and SK609 from 5 to 60 min. Further, the ratio of pDAT to tDAT (pDAT/tDAT) expression (Figure 1D) paralleled the differences observed in pDAT expression between treatments. A two-way repeated measures ANOVA revealed a significant effect of treatment [F­(7, 32) = 44.06, *p* < 0.001], time [F­(7, 32) = 335.1, *p* < 0.001], and a treatment × time interaction [F­(7, 32) = 23.54, *p* < 0.001] for pDAT/tDAT expression. Šidák posthoc tests revealed significant differences between PRX and SK609 from 5 to 60 min.

**Figure 1:**
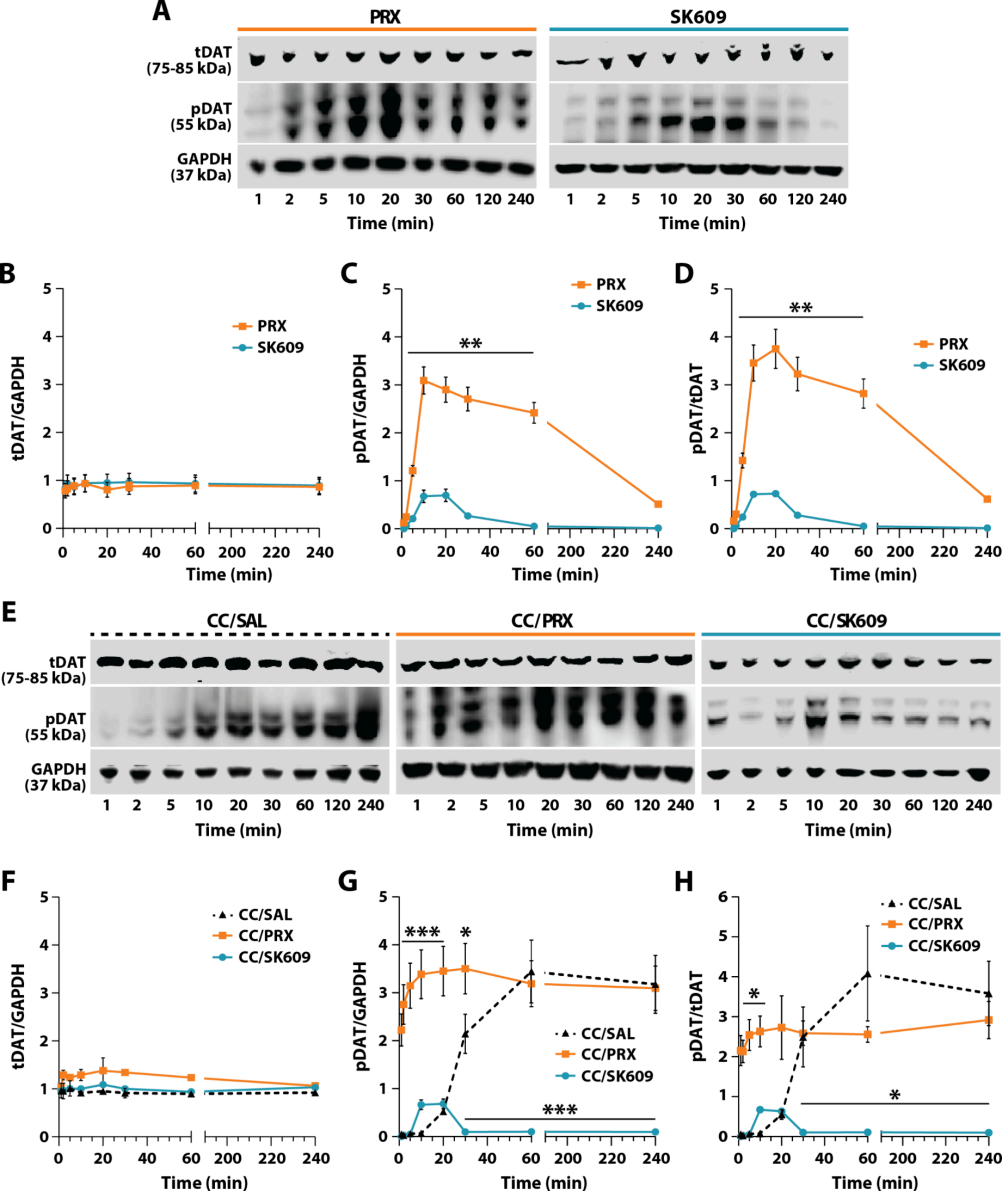
Temporal activation of DAT by D3R agonists *in vitro*. Time course profiles of phosphorylated DAT (pDAT) and total DAT (tDAT) expression from SH-SY5Y-D3R cells treated with D3R agonists with cocaine (CC) and without CC pretreatment. (A) Representative Western blots for tDAT, pDAT, and GAPDH (loading control). Quantification of (B) tDAT/GAPDH, (C) pDAT/GAPDH, and (D) pDAT/tDAT. (E) Representative Western blots for tDAT, pDAT, and GAPDH with cocaine pretreatment. Quantification of (F) tDAT/GAPDH, (G) pDAT/GAPDH, and (H) pDAT/tDAT. Data are represented as mean ± SEM (B–D) Šidák tests; **p < 0.01 PRX vs SK609. (F–H) Dunnett’s tests; *p < 0.05, ***p < 0.001 vs CC/SAL.

To determine if cocaine exposure impacted D3R biased signaling effects on DAT expression and phosphorylation, SH-SY5Y-D3R cells were pre-exposed to cocaine for 5 min in media prior to treatment with SAL, PRX, or SK609 (Figure 1E). Cocaine pretreatment resulted in minimal changes in tDAT expression relative to GAPDH for all treatments (Figure 1F). A two-way ANOVA revealed no effect of treatment [F­(2, 6) = 3.901, *p* = 0.082], time [F­(2.2623, 15.74) = 1.260, *p* = 0.319], or a treatment × time interaction [F­(13, 42) = 0.9497, *p* = 0.517] on tDAT expression. Exposure to cocaine/saline (*n* = 3) and cocaine/PRX (*n* = 3) promoted a sustained increase in pDAT expression, while cocaine/SK609 treatment (*n* = 3) resulted in significantly lower pDAT expression, with activation lasting for only 30 min (Figure 1G). A two-way ANOVA for pDAT expression revealed significant effects of treatment [F­(2, 48) = 149.2, *p* < 0.001], time [F­(7, 48) = 8.111, *p* < 0.001], and a treatment × time interaction [F­(13, 48) = 6.762, *p* < 0.001]. Dunnett’s posthoc tests revealed significant differences between cocaine/saline and cocaine/PRX for the first 1–30 min as well as between cocaine/saline and cocaine/SK609 treatment from 30 to 240 min. Additionally, pDAT/tDAT paralleled trends observed between treatments for pDAT expression. A two-way ANOVA for pDAT/tDAT revealed significant effects of treatment [F­(2, 6) = 14.63, *p* = 0.005], time [F­(1.620, 9.718) = 12.37, *p* = 0.003], and treatment × time interaction [F­(14, 42) = 10.71, *p* < 0.001]. Dunnett’s posthoc tests showed significant differences between cocaine/saline and cocaine/PRX for the first 1–10 min post-treatment and between cocaine/saline and cocaine/SK609 at 10 min following treatment (Figure 1H). Together, these results indicate that PRX augments the cocaine-associated phosphorylation of DAT, while SK609 reduces this phosphorylation. These results are consistent with previous studies that showed acute treatment with unbiased D3R agonists increased phosphorylated DAT in HEK cells.^65^

SK609 treatment largely blocked the effects of cocaine, indicating that G-protein biased D3R activation prevents cocaine-induced phosphorylation of the DAT. This result is consistent with our hypotheses, given that G-protein biased D3R activation recruits inhibitory G_i_-coupled signaling pathways. The observed increase and prolonged expression of pDAT with cocaine/PRX exposure is likely due to the long-term ERK1/2 signaling through β-arrestin2 recruitment, as we have previously published.^59^ These effects of PRX may be synergistic with cocaine effects on ERK1/2, resulting in increased phosphorylation of DAT at Thr53.^66,67^ It is also notable that the synergistic effect of cocaine/PRX is observed only with pDAT expression and not tDAT as indicated by the pDAT/tDAT ratio, suggesting the potentiation of pDAT is not due to an increase in the total number of transporters.

### PRX Increased Striatal pDAT Expression in Rats That Received Cocaine

To examine the effects of PRX and SK609 on pDAT expression *in vivo* with and without cocaine exposure, male Sprague–Dawley rats received either pretreatment injections of saline 15 min prior to receiving injections of saline (*n* = 5), PRX (*n* = 5; 0.25 mg/kg), or SK609 (*n* = 3; 4 mg/kg) or a pretreatment injection of cocaine (10 mg/kg) 15 min prior to injection of saline (*n* = 6), PRX (*n* = 3), or SK609 (*n* = 6) for 7 days (1x/day; i.p.). Drug doses were chosen based on previous studies using SK609,^68–70^ PRX,^53,71,72^ or cocaine.^73–75^ The whole striatal tissue was dissected on the final day for Western blotting and subsequent densiometric analysis (Figure 2A). There were no changes in the expression of tDAT or pDAT in saline-pretreated rats following PRX or SK609. Notably, rats treated with cocaine alone (cocaine/saline) did not exhibit changes in tDAT or pDAT expression, while PRX increased pDAT following cocaine pretreatment (cocaine/PRX) relative to controls (saline/saline). By comparison, SK609 did not increase pDAT expression (cocaine/SK609), suggesting that recruitment of β-arrestin2 by PRX promoted the observed increase in pDAT with cocaine pretreatment. A two-way ANOVA for tDAT expression revealed no significant effect of pretreatment (i.e., saline or cocaine) [F­(1, 22) = 0.4348, *p* = 0.516], treatment (i.e., saline vehicle, PRX, or SK609) [F­(2, 22) = 0.02941, *p* = 0.971], or a pretreatment × treatment interaction [F­(2, 22) = 0.04912, *p* = 0.952] (Figure 2B). However, a two-way ANOVA for pDAT expression revealed a significant effect of treatment [F­(2, 22)=7.394, *p* = 0.003] with no significant effect of pretreatment [F­(1, 22) = 0.0006274, *p* = 0.980] or a pretreatment × treatment interaction [F­(2, 22) = 1.666, *p* = 0.212]. Dunnett’s posthoc tests demonstrated a significant difference in pDAT expression between saline/saline controls and cocaine/PRX (Figure 2C). Finally, a two-way ANOVA for pDAT/tDAT expression revealed a significant effect of treatment [F­(2, 22) = 8.054, *p* = 0.002] but no significant effect of pretreatment [F­(1, 22) = 0.5200, *p* = 0.478] or a pretreatment × treatment interaction [F­(2, 22) = 1.756, *p* = 0.196]. Dunnett’s posthoc tests demonstrated a significant difference between saline/saline controls and cocaine/PRX (Figure 2D).

**Figure 2:**
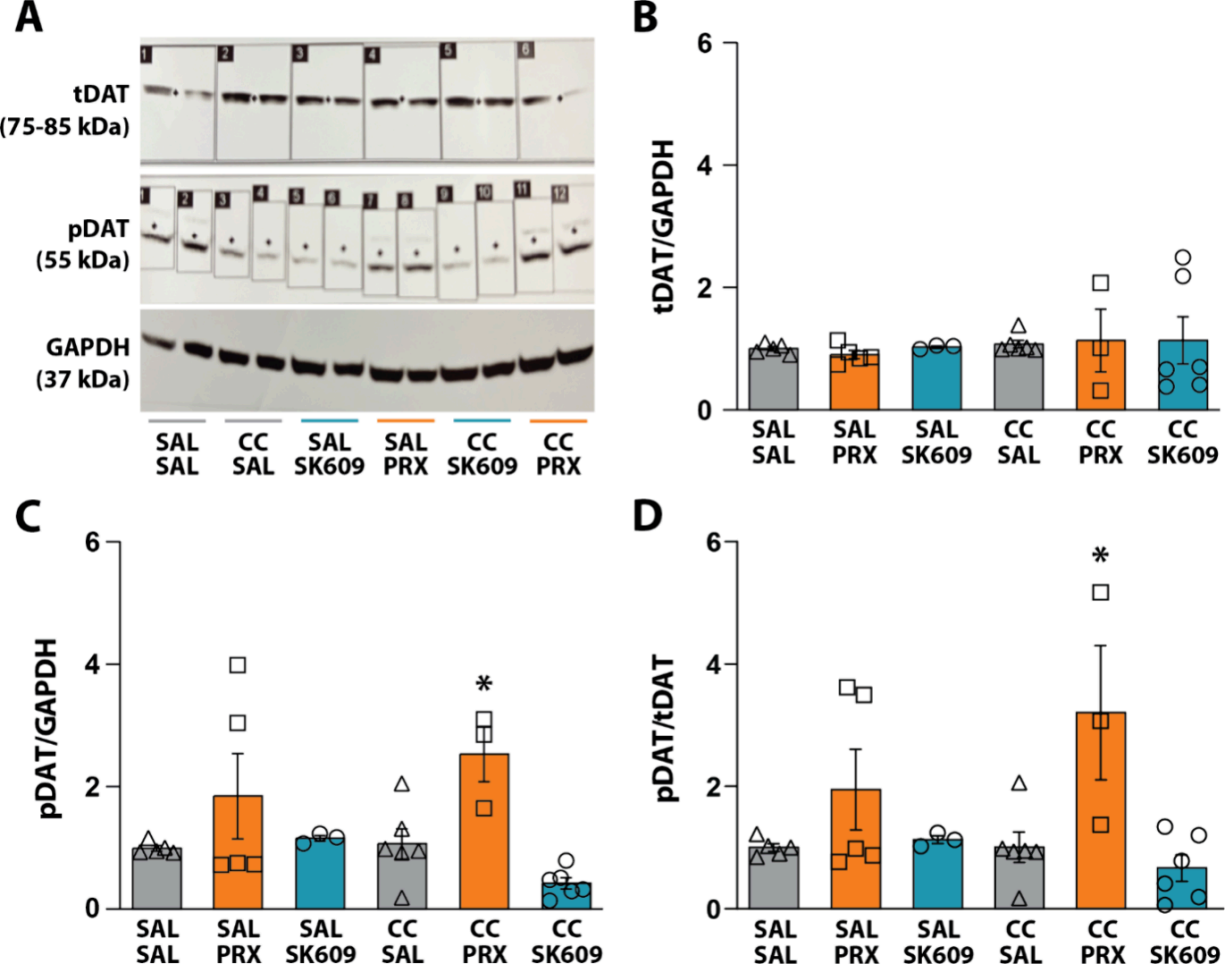
Effects of *in vivo* D3R agonists on DAT expression and phosphorylation without and following cocaine exposure. Male, Sprague–Dawley rats received ip pretreatment of either saline (SAL) or cocaine (CC) 15 min prior to treatment with saline (SAL/SAL *n* = 5; CC/SAL *n* = 6), PRX (0.25 mg/kg; SAL/PRX *n* = 5; CC/PRX *n* = 3), or SK609 (4 mg/kg; SAL/SK609 *n* = 3; CC/SK609 *n* = 6) 1x/day for 7 days. (A) Representative Western blots for total DAT (tDAT), phosphorylated DAT (pDAT), and GAPDH. Quantification of (B) tDAT/GAPDH, (C) pDAT/GAPDH, and (D) pDAT/tDAT. Data are represented as mean ± SEM normalized to SAL/SAL control. (B-D) Dunnett’s tests; *p < 0.05 vs SAL/SAL.

These observations suggest that repeated *in vivo* treatment with PRX potentiates the effects of cocaine by upregulating pDAT expression in the striatum, while the same effect is not observed with SK609. These findings are consistent with the observed *in vitro* effects of PRX and SK609 (Figure 1) and with studies in mice that reported that chronic treatment with PRX promoted increased interactions of D3R with DAT.^41^ The distinct effects on pDAT and tDAT expression following both unbiased and G-protein biased treatments suggest that PRX and SK609 specifically impact pDAT sensitivity to cocaine (i.e., pDAT levels) without changing the number of transporters, as quantified by pDAT/tDAT expression (Figure 2D).

One potential mechanism by which D3R activation may influence DAT expression and DA transmission presynaptically is via direct physical interaction. For example, D3R activation induces DAT internalization which subsequently reduces DA uptake.^41,60–63^ Alternatively, it is possible that the effects of D3R activation and DA transmission are mediated by D3R expressed on cholinergic interneurons in the NAc, which influence DA release via activation of nicotinic acetylcholine receptors on DA terminals.^18,63,76–79^ Future studies will be needed to determine to what degree the effects of PRX and SK609 on DAT function involve direct presynaptic interactions versus interactions with cholinergic interneurons.

### PRX and SK609 Differentially Affect DAT Function in NAc Core of Naive Rats

To determine if systemic administration of PRX and SK609 exerts differential effects on DA transmission, we used *ex vivo* FSCV to measure DA peak concentrations (DA peak height) and DA uptake. Given that our prior studies on DAT function were focused within the NAc core,^10,80–83^ the present recordings were collected from the NAc core in slices from naive rats treated with saline (*n* = 8), PRX (*n* = 8), or SK609 (*n* = 8). As shown in averaged DA signal traces (Figure 3A,B), while neither PRX nor SK609 significantly influenced DA peak height, SK609 significantly reduced DA uptake. One-way ANOVAs revealed a significant effect of treatment on DA peak height [F­(2, 21) = 4.588, *p* = 0.0222] (Figure 3B) and a significant effect of treatment on DA uptake [F­(2, 20)=6.794, *p* = 0.0056] (Figure 3C). While Dunnett’s posthoc tests did not reveal any group differences for DA peak height, these tests revealed a significant difference in DA uptake between saline and SK609. These findings indicate that PRX and SK609 differentially affect DAT function with SK609 decreasing DA uptake.

**Figure 3:**
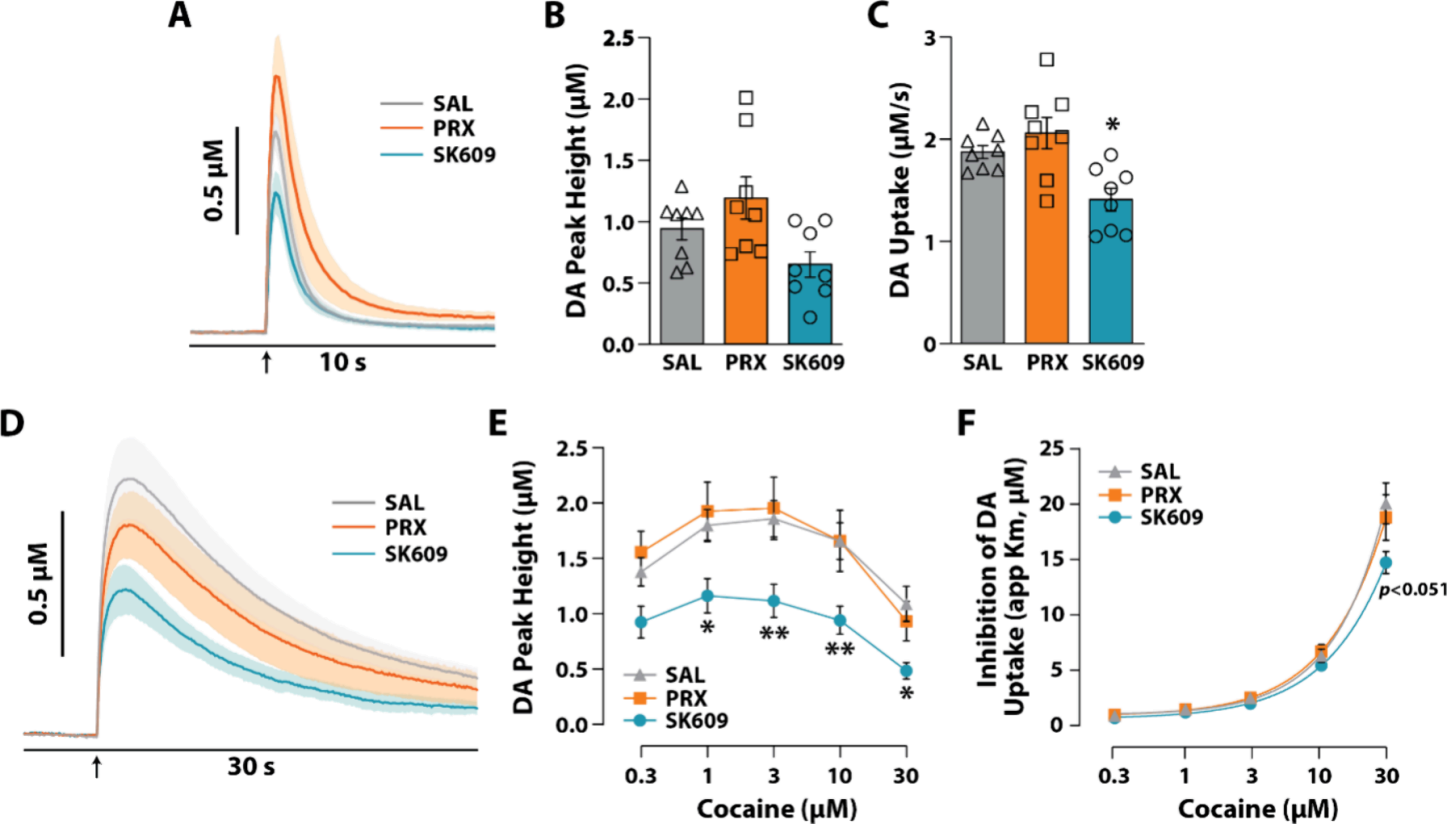
Acute *ex vivo* effects of D3R agonists on DAT dynamics in the NAc core. Male, Sprague–Dawley rats received an i.p. injection of saline (SAL, *n* = 8), (PRX, *n* = 8), or (SK609, *n* = 8) 30 min prior to euthanasia for FSCV. (A) Average baseline DA traces are depicted by solid lines with SEM represented as shading. (B) DA peak height (μM) and (C) DA uptake (μM/s, Vmax). (D) Average DA traces for 30 μM cocaine are depicted by solid lines with the SEM represented as shading. (E) DA peak height (μM) and (F) inhibition of DA uptake (μM) are quantified for each cocaine concentration. (A, D) Arrow depicts time of electrical stimulation. Data are represented as mean ± SEM (E, F) Dunnet’s tests; *p < 0.05, **p < 0.01 vs SAL.

We next examined whether PRX and SK609 influenced the effects of cocaine on DA peak height and inhibition of DA uptake at increasing concentrations of cocaine. We observed that cocaine significantly increased DA peak height following saline and PRX, but that SK609 significantly attenuated the effects of cocaine (Figure 3E). A two-way repeated measures ANOVA revealed a significant effect of treatment [F­(2, 21) = 4.914, *p* = 0.018] and of cocaine concentration [F­(2.032, 42.67) = 66.46, *p* < 0.001] but not a treatment × concentration interaction [F­(8, 84) = 1.779, *p* = 0.093] on DA peak height (Figure 3D). Dunnett’s posthoc tests revealed significant differences in DA peak height between saline and SK609 at 1, 3, 10, and 30 μM cocaine but no difference between saline and PRX at any concentration. We then examined to what extent PRX and SK609 influenced DAT sensitivity to cocaine (i.e., cocaine-induced inhibition of DA uptake) and observed a modest decrease in DAT sensitivity to cocaine following SK609 (Figure 3E). A two-way repeated measures ANOVA demonstrated a significant effect of concentration [F­(1.166, 24.49) = 256.0, *p* < 0.001] and a significant treatment × concentration interaction [F­(8, 84) = 2.216, *p* = 0.034] with a nearly significant effect of treatment [F­(2, 21) = 3.350, *p* = 0.055] on inhibition of DA uptake (Figure 3E). A Dunnett’s posthoc tests revealed a strong trend (*p* = 0.051) for a difference between saline and SK609 at 30 μM cocaine, suggesting that SK609 may be reducing the effects of cocaine on inhibition of DA uptake.

Together, these results demonstrate that G-protein biased D3R activation by SK609 reduces DA uptake under baseline conditions and attenuates the effects of cocaine on DA peak height. This is likely due to reduced pDAT levels within NAc (Figure 2) and the subsequent trend for reduced DAT sensitivity to cocaine. Consistent with this, we previously demonstrated that activation of G_i_ DREADDs in ventral tegmental area DA neurons reduced the effects of cocaine on DA uptake and pDAT expression in the NAc.^11^ While both PRX and SK609 signal through inhibitory G-protein coupled receptors, which we expect would lead to disruptions in DA transmission, the additional recruitment of β-arrestin2 by PRX may counteract this inhibitory G-protein signaling effect, thereby impeding the effects of cocaine on DA transmission.

### PRX Significantly Increased Cue-Induced Cocaine Seeking

Given the observation that SK609 and PRX altered DA transmission, we examined to what extent D3R agonist signaling impacts cocaine seeking^64,80,84,85^ – a behavior known to be mediated by NAc DA dynamics.^86–88^ Male Long Evans rats underwent intermittent access (IntA) to cocaine and were tested on cue-induced seeking tests on abstinence days (AD) 1 and AD7. Rats were treated with saline (*n* = 7), PRX (*n* = 6), or SK609 (*n* = 6) daily from AD2 to AD7 (Figure 4A). We first validated that there were no differences in self-administration prior to treatment group assignment by assessing the number of days required to meet long access (LgA) acquisition criteria (Figure 4B) and the average number of lever presses during the last 2 days of acquisition on LgA (Figure 4C). As anticipated, no group differences were observed for these measures. A one-way ANOVA revealed no differences in days to acquire between groups [F­(2, 18) = 1.150, *p* = 0.3389] (Figure 4B). Further, a two-way ANOVA revealed no significant effect of group on lever presses during the last 2 days of acquisition [F­(2, 41) = 0.5226; *p* = 0.597] or a group × lever type interaction [F­(2, 1) = 0.9874; *p* = 0.381] (Figure 4C). However, there was a significant effect of lever type (active versus inactive lever presses) [F­(1, 41) = 90.38; *p* < 0.001], thereby demonstrating sufficient differentiation between active and inactive levers (Figure 4C).

**Figure 4:**
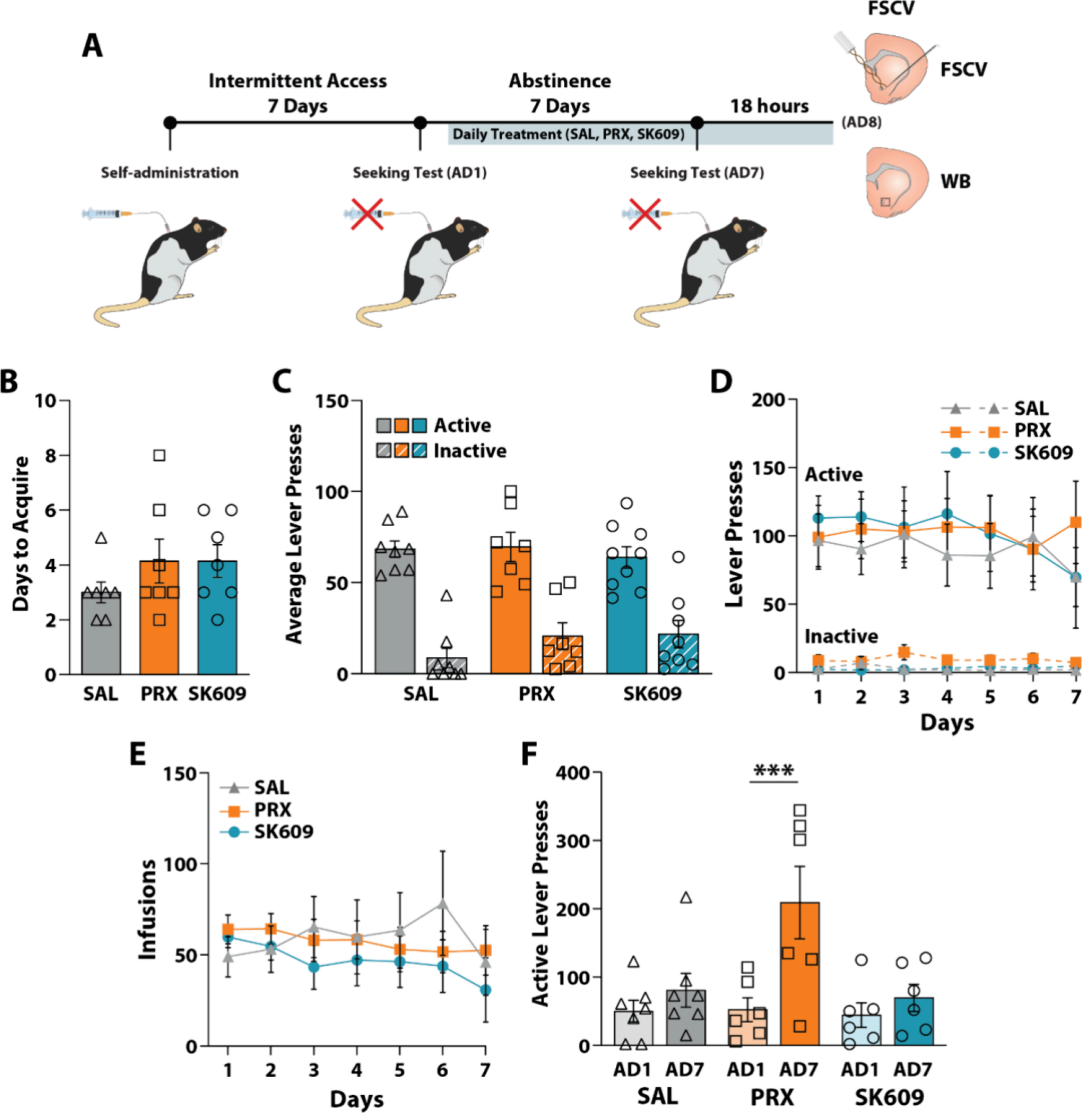
Effects of D3R agonists on cue-induced cocaine seeking after repeated treatment during forced abstinence. Male, Long Evans rats were treated with saline (SAL; *n* = 7), pramipexole (PRX, 0.25 mg/kg; *n* = 6), or SK609 (4 mg/kg; *n* = 6) beginning on abstinence day (AD) 2. (A) Experimental timeline. (B) Average number of days to acquire self-administration behavior and (C) average active and inactive lever presses for the last two sessions of acquisition. (D) Average active and inactive lever presses as well as (E) average infusions during IntA self-administration across each session. (F) Cue-induced seeking was measured 24 h (AD1) and 7 days (AD7) into abstinence as the number of active lever presses within 1 h seeking tests. Data presented are mean ± SEM (D) Dunnett’s tests; ***p < 0.001 vs AD1. (WB, Western blot).

With respect to IntA sessions, we observed no significant differences in lever presses (Figure 4D) or infusions (Figure 4E), further validating that there were no group differences prior to group assignment. A mixed-effects model revealed no effect of group [F­(2, 21) = 0.09956, *p* = 0.906], session [F­(2.747, 56.31) = 1.171, *p* = 0.327], or a group × session interaction [F­(12, 123) = 0.6271, *p* = 0.816] on active lever pressing during IntA. Similarly, a mixed-effects model for inactive lever presses revealed no significant effect of session [F­(3.120, 63.97) = 0.2891, *p* = 0.841] or a group × session interaction [F­(12, 123) = 1.031, *p* = 0.425], though there was a significant effect of group [F­(2, 21) = 5.496], *p* = 0.012]. Note that this difference in inactive lever presses has no bearing on the amount of cocaine consumed during self-administration. Importantly, a mixed-effects model showed no significant effects of group [F­(3, 36) = 0.2641, *p* = 0.851], session [F­(1.807, 64.43) = 1.833, *p* = 0.172], or a group × session interaction [F­(7, 214) = 0.7139, *p* = 0.795] on IntA infusions, indicating that cocaine consumption did not differ between groups prior to treatment.

We next examined the effects of PRX and SK609 on cue-induced cocaine seeking and observed a robust increase in cue-induced seeking with PRX but not with SK609. A two-way repeated measures ANOVA revealed a significant effect of abstinence day [F­(1, 16) = 13.99, *p* = 0.002], a treatment × abstinence day interaction [F­(2, 16) = 5.023, *p* = 0.020], and a trend for a treatment effect on active lever presses [F­(2, 16) = 3.275, *p* = 0.064]. Dunnett’s posthoc tests revealed a significant increase in active lever presses between AD1 and AD7 following PRX treatment with no increase observed following saline or SK609. These observations are consistent with clinical and preclinical studies indicating that PRX exerts rewarding/reinforcing properties itself,^53,89,90^ promotes impulsivity,^91,92^ and enhances the reinforcing effects of cocaine.^53,93,94^ Moreover, these findings suggest that recruitment of β-arrestin2 by PRX potentiates cocaine seeking, an effect that is absent following SK609 which does not recruit β-arrestin2.

In the present study, we sought to determine the effects of D3R biased signaling early in the abstinence period. Based on our recent report,^95^ we anticipated observing a modest, albeit significant, increase in cocaine seeking in saline-treated rats on AD7. Further, we hypothesized that PRX would increase cocaine seeking, while SK609 would decrease seeking compared to that of saline-treated rats. While we observed a robust increase in cocaine seeking following PRX treatment, the absence of increased cocaine seeking in saline-treated rats on AD7 makes it difficult to determine whether SK609 may exert reductions in cocaine seeking. Therefore, despite potential beneficial effects of SK609 on pDAT expression and DA transmission observed *in vitro* and, in the *ex vivo* FSCV experiments in naive rats (Figures 1 and 3), the present findings indicate that SK609 does not reduce cocaine seeking under conditions where control rats display low seeking themselves.

Previous studies have demonstrated that extending abstinence to longer time periods (e.g., 28 days) is associated with greater cocaine seeking, as previously established for the incubation of cocaine seeking model.^80,96,97^ Therefore, future studies would benefit from extending the abstinence period (e.g., 14, 21, or 28 days) to determine the effects of PRX and SK609 under conditions of robust incubation of cocaine seeking.

### Repeated Treatment with PRX Increased pDAT Expression during Abstinence

NAc tissue from a subset of the rats that underwent self-administration behavior (see Figure 4A timeline) was dissected on AD8 for Western blotting of tDAT and pDAT quantified relative to GAPDH expression. Repeated treatment with saline (*n* = 4), PRX (*n* = 4), or SK609 (*n* = 4) had no effect on the expression of tDAT (Figure 5A,B), while PRX significantly increased pDAT expression (Figure 5A,C). A one-way ANOVA revealed no effect of treatment on tDAT expression [F­(2, 10) = 1.853, *p* = 0.2118] (Figure 5B). However, one-way ANOVAs revealed a significant effect of treatment on pDAT [F­(2, 10) = 4.784, *p* = 0.0384] (Figure 5C) and pDAT/tDAT [F­(2, 10) = 9.472, *p* = 0.0061] expression (Figure 5D). Dunnett’s posthoc analyses demonstrated a significant difference in pDAT/tDAT between saline and PRX. These data indicate that repeated PRX treatment during abstinence augments pDAT expression, thereby suggesting that increased phosphorylation at Thr53 may contribute to the increased cocaine seeking observed. This observation is consistent with prior studies demonstrating that elevations in pDAT expression are associated with increased cocaine seeking.^11,12^

**Figure 5:**
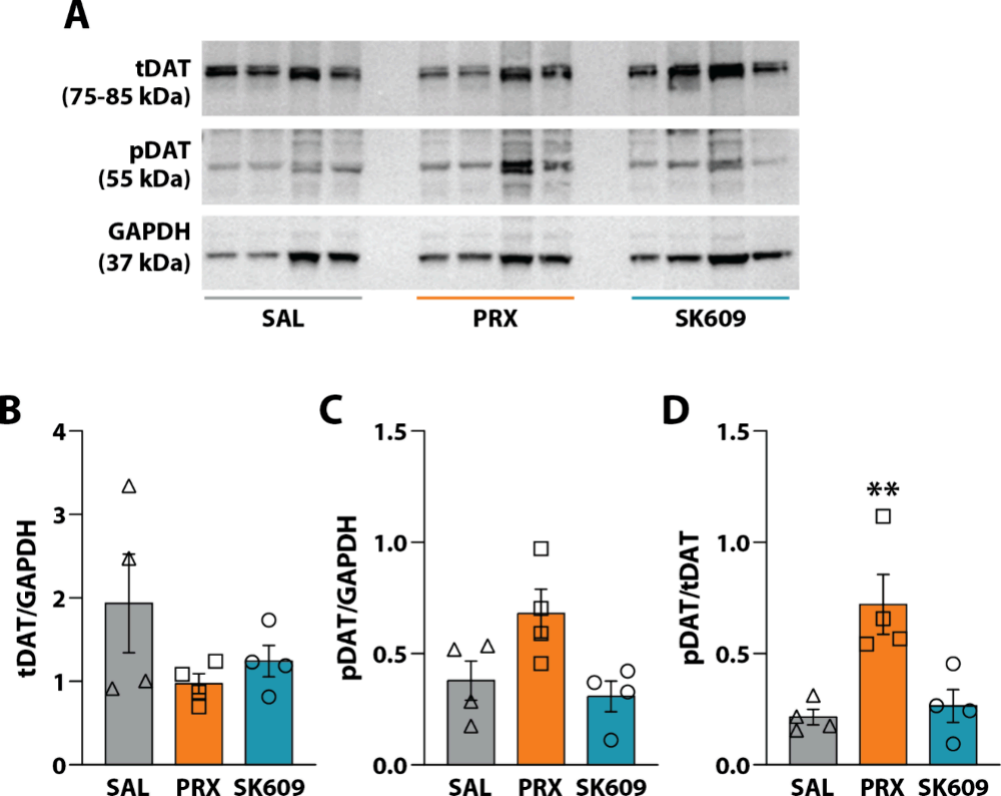
Effects of repeated D3R agonist treatment *in vivo* on DAT expression and phosphorylation during abstinence from cocaine. Male, Long Evans rats were treated with saline (SAL; *n* = 4), pramipexole (PRX, 0.25 mg/kg; *n* = 4), or SK609 (4 mg/kg, *n* = 4) on abstinence day (AD) 2 through AD8. (A) Representative Western blots for total DAT (tDAT), phosphorylated DAT (pDAT), and GAPDH (loading control) used NAc tissue collected on AD8. Quantification of (B) tDAT/GAPDH, (C) pDAT/GAPDH, and (D) pDAT/tDAT. Data are represented as mean ± SEM. Dunnett’s tests; **p < 0.05 vs SAL.

Although SK609 reduced pDAT *in vitro* and acutely disrupted DA transmission in naive rats, it had no effect on pDAT expression following 7 days of abstinence from IntA to cocaine. SK609 has high distribution in the rat striatum and a half-life of >4 h, suggesting that this lack of effect is not due to low drug concentrations in the brain.^58,69^ Considering our behavioral findings, this lack of effect on pDAT expression compared to saline-treated rats is also not surprising, given that neither of these groups displayed changes in cocaine seeking over the 7 day abstinence period.

### PRX Enhances the Effects of Cocaine on DA Peak Height Following Abstinence from Cocaine

To assess if the effects of PRX on cocaine seeking are associated with changes in DA transmission within the NAc core, we performed *ex vivo* FSCV in the same rats tested for cue-induced seeking (see the Figure 4A timeline). The day following the AD7 seeking test, rats were treated with saline (*n* = 7), PRX (*n* = 6), or SK609 (*n* = 6) 30 min prior to preparing NAc slices for FSCV (Figure 6). Under baseline conditions (i.e., no cocaine present), we observed no significant differences in DA peak height (Figure 6A,B) or DA uptake (Figure 6A,C). One-way ANOVAs for baseline DA peak height [F­(2, 15) = 1.149, *p* = 0.3434] and DA uptake [F­(2, 16) = 1.466, *p* = 0.2621] revealed no significant effects of treatment.

**Figure 6:**
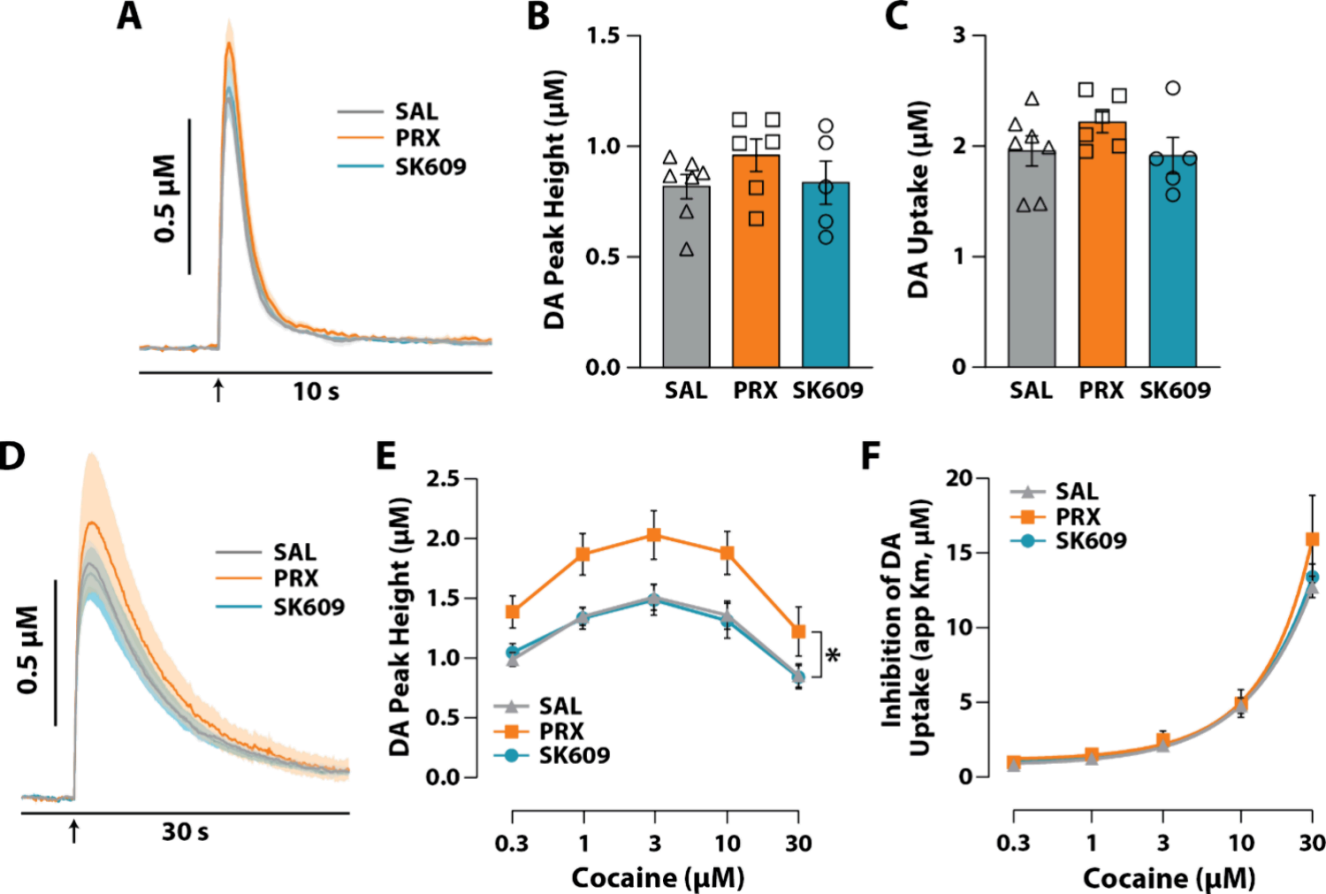
Effects of repeated D3R agonist treatment on*ex vivo*DAT dynamics in the NAc core. Male, Long Evans rats were treated with saline (SAL; *n* = 7), pramipexole (PRX, 0.25 mg/kg; *n* = 6), or SK609 (4 mg/kg; *n* = 6) beginning on abstinence day (AD) 2 and through AD8. (A) Average DA traces for baseline recordings (i.e., no cocaine) are represented by the solid line with SEM presented as shading. (B) DA peak height (μM) and (C) DA uptake (μM/s, *V*_max_). (D) Average DA traces for 30 μM cocaine are represented by the solid line with the SEM presented as shading. (E) DA peak height (μM) and (F) inhibition of DA uptake (μM). (A,D) Arrow depicts time of electrical stimulation. Data are represented as mean ± SEM. (D) Two-way repeated measures ANOVA, main effect of treatment; *p < 0.05.

Notably, we observed that PRX enhanced the effects of cocaine on the DA peak height (Figure 6D,E). A two-way repeated measures ANOVA revealed a significant effect of cocaine concentration [F­(1.761, 24.65) = 60.57, *p* < 0.001] and treatment [F­(2, 14) = 5.360, *p* = 0.019] but there was no concentration × treatment interaction [F­(8, 56) = 0.6482, *p* = 0.734] on DA peak height (Figure 6E). However, Dunnett’s posthoc tests did not reveal any significant differences between treatment groups at specific time points. For inhibition of DA uptake, a two-way repeated measures ANOVA revealed a significant effect of cocaine concentration [F­(1.309, 18.32) = 182.9, *p* < 0.001] but no significant effect of treatment [F­(2, 14) = 0.6881, *p* = 0.519] and no concentration × treatment interaction [F­(8, 56) = 1.034, *p* = 0.422] (Figure 6F). When considered together, these results indicate that daily PRX treatment during abstinence augmented the effects of cocaine on the DA peak height, suggesting that recruitment of β-arrestin2 may contribute to these effects. Further, these findings suggest that the observed increases in cocaine seeking on AD7 (Figure 4F) following PRX treatment may be linked to increased pDAT expression (Figure 5) and related changes in DA dynamics (Figure 6).

SK609 treatment during abstinence did not affect either cocaine seeking (Figure 4F) or pDAT expression (Figure 5) relative to saline-treated rats. Consistent with this, SK609 did not affect the DA peak height or DA uptake. As discussed above, this lack of SK609 effect following abstinence from IntA to cocaine may be related to the early abstinence time point tested herein. Follow up studies will examine whether a longer period of abstinence reveals potential beneficial effects of SK609 on DAT expression, function, and related behavior.

## Methods

### Materials

All cell culture reagents were purchased from Invitrogen (Carlsbad, CA), and protease inhibitor cocktail, phosphatase inhibitor cocktail, Restore PLUS stripping buffer, and lysis buffer were obtained from Thermo Scientific (Rockford, IL). G418 was purchased from Gemini Bio-Products (West Sacramento, CA). Pramipexole (PRX) and puromycin were obtained from Sigma-Aldrich (St. Louis, MO), and SK609 was synthesized in-house as previously described.^58^ Cocaine was obtained from the National Institute on Drug Abuse drug supply program in accordance with the United States Drug Enforcement Administration regulation guidelines. The drug doses used were chosen based on our previous studies on SK609 where peak efficacy was observed at 4 mg/kg.^68–70^ We used a clinically relevant dose for PRX (0.25 mg/kg),^71,72^ which is low enough to preferentially activate D3Rs with minimal D2R effects and cocaine doses and concentrations chosen were based on prior studies.^73–75,80^

### Cell Culture

SH-SY5Y cells stably expressing human D3R (SH-SY5Y-D3R) by pEGFP-N1 plasmid vector were grown in 100 mm culture dishes in Dulbecco’s modified Eagle medium (DMEM) supplemented with 10% fetal calf serum, 100 units/mL penicillin and 100 μg/mL streptomycin in a humidified 5% CO2 atmosphere at 37 °C.^58^ SY-SY5Y-D3R cells were grown in the presence of 0.1 mg/mL G418 to maintain stable D3R expression.

### Time Course Activation Assay

SH-SY5Y-D3R cells (0.5 × 10^6^) were seeded in six-well plates, cultured in 3 mL of DMEM complete growth medium for 24 h, and then incubated with 3 mL of DMEM without serum for 2 h. Cocaine (10 mM) or phosphate-buffered saline (PBS) was added to the cells and then treated with either PRX (5 nM) or SK609 (1 μM) and incubated for the indicated times (1, 2, 5, 10, 20, 30, 60, and 240 min) at 37 °C as previously described.^59^ The doses of drugs (10xEC50 value at D3R) were chosen based on our previous studies.^59^ The reaction was stopped by aspirating the medium and washing the cells with 1 mL of PBS followed by the addition of 100 μL/well lysis buffer with 1X Protease Inhibitor Cocktail, 1X Phosphatase Inhibitor Cocktail, and 1 mM PMSF (phenylmethylsulfonyl fluoride) to solubilize cells.

### Western Blotting of SH-SY5Y-D3R Cell Lysates

Cell lysates collected from the time course activation assay were assessed for protein content using the DC Protein Assay Kit II (Bio-Rad) with BSA as a standard. Loading buffer was added to protein samples, boiled for 5 min at 100 °C in a water bath, and spun at 2500*g*. 20 μg total protein/lane was loaded to SurePAGE, 4–12% Bis-Tris Mini Gel for separation (GenScript) for 2 h and transferred to a PVDF (polyvinylidene fluoride) membrane (Thermo Scientific).

Immunoblotting was performed with rabbit antiphospho-Thr53 DAT polyclonal antibody (1:1000, PhosphoSolution) and peroxidase-conjugated goat anti-rabbit IgG (H+L) (1:5000, Jackson Immuno Research Laboratories, Inc.). In the same blots, total DAT (tDAT) and GAPDH levels were also determined with rabbit anti DAT polyclonal antibody (1:1000, EMD Millipore Corp) and rabbit anti GAPDH (1:5000, Invitrogen), respectively, after stripping the blots. Chemiluminescence detection was performed using the SuperSignal West Dura Extended Duration Substrate detection kit (Thermo Scientific). Both pDAT and tDAT immunoblots were quantified by densitometry with an ImageQuant LAS4000 (GE Healthcare Biosciences, Pittsburgh, PA) and normalized to GAPDH expression levels.

### Animals

Adult male Sprague–Dawley (naive experiments) and Long Evans (behavioral experiments) rats (250–350 g, Envigo) were housed under reverse light cycle (12:12) conditions at 24 °C. All rats received food and water ad libitum and were separated from paired housing following surgery. All protocols were conducted in accordance with the National Institutes of Health Guide for the Care and Use of Laboratory Animals under the supervision of the Institutional Animal Care and Use Committee at Drexel University.

### Tissue Dissection

To dissect brain tissue for fast scan cyclic voltammetry (FSCV) and Western blots, rats were anesthetized for 5 min with 2.5% isoflurane 30 min after intraperitoneal (i.p.) injection of either saline vehicle (SAL), PRX (0.25 mg/kg), or SK609 (4 mg/kg) for rapid decapitation and whole-brain extraction. All tools were sterile or sanitized with 70% isopropyl alcohol prep wipes (McKesson). The anterior portion of the brain was placed in ice-cold, oxygenated artificial cerebrospinal fluid (aCSF; NaCl (126 mM), KCl (2.5 mM), NaH_2_PO_4_ (1.2 mM), CaCl_2_ (2.4 mM), MgCl_2_ (1.2 mM), NaHCO_3_ (25 mM), glucose (11 mM), and l-ascorbic acid (0.4 mM) in ultrapure water). Striatal sections (400 μm) containing the NAc were collected using a vibrating microtome, cut at the midline, and then allowed to equilibrate for 1 h in oxygenated aCSF at room temperature. One slice was used for FSCV experiments to record dopamine dynamics in the NAc core, and NAc tissue was dissected from the remaining slices for Western blotting (stored in RNAlater at 4̊ °C or at −80̊ °C until protein extraction).

### *Ex Vivo* Fast Scan Cyclic Voltammetry

Rats were euthanized, and 400 μM thick tissue sections containing the NAc core were collected for FSCV as previously described.^80,81,98^ Briefly, a carbon-fiber microelectrode was placed between a bipolar stimulating electrode (Plastics One, Roanoke, VA) positioned on the surface of the NAc core. DA release was stimulated with a single monophasic electrical pulse (400 μA, 4 ms) every 3 min and recorded by applying a triangular waveform (−0.4 to +1.2 to −0.4 V) at a scan rate of 400 V/s using Demon Voltammetry and Analysis software.^99^ Dopamine peak height (μM DA), maximal DA uptake rate (*V*_max_), and inhibition of DA uptake (apparent *K*_m_) were modeled using Michaelis–Menten kinetics. Rats were injected i.p. with saline, PRX (0.25 mg/kg), or SK609 (4 mg/kg) 30 min prior to being euthanized for FSCV to determine *in vivo* effects of the compounds on DA transmission in the NAc. All rats were kept in the dark for 30 min prior to euthanasia. Only one 400 μm hemisphere slice was used for FSCV recordings for each rat, and the remainder (approximately 3–5 hemisphere slices) were processed for Western blotting.

### Western Blotting of Striatal or NAc Tissue

To examine the effects of D3R agonists PRX and SK609 on pDAT and tDAT expression, Western blotting was performed as described above in total striatal (Figure 2) or ventral striatal NAc tissue (Figure 5). For all rats, tissue was collected from rats that received an i.p. injection of either saline, PRX (0.25 mg/kg), or SK609 (4 mg/kg) 30 min prior to euthanasia. Rats from which total striatal tissue was dissected for Western blotting (Figure 2) received seven daily i.p. injections of either saline or cocaine (10 mg/kg) followed by the treatments (saline vehicle, PRX at 0.25 mg/kg, or SK609 at 4 mg/kg) 15 min later. Additionally, after the last treatment on day seven, all striatal tissue (2 mm thick section) was rapidly dissected on ice, weighed, homogenized in ice-cold lysis buffer, and centrifuged for 5 min at 4 °C. The supernatant fraction was re-centrifuged and the resulting crude synaptosome fraction was collected.^100^ Immunoblotting for tDAT, pDAT, and GAPDH was performed as described previously for cell lysates. Both pDAT and tDAT immunoblots were quantified by densitometry with ImageJ (NIH) and normalized to GAPDH expression. For injected rats that did not self-administer cocaine (Figure 2), all immunoblots were normalized to the saline/saline control group.

### Surgical Procedures

Rats underwent jugular vein catheterization surgeries as previously described.^80^ Briefly, an intravenous catheter (3 Fr) was inserted into the right jugular vein and tethered to an access port with magnetic connectors (Instec Laboratories, Plymouth Meeting, PA) secured into the skin caudal to the scapulae. All rats received peri- and postoperative subcutaneous injections of Ketoprofen (5 mg/kg; Patterson Veterinary, Devens, MA) and Enrofloxacin (5 mg/kg; Norbrook, Northern Ireland) in addition to application of a combined antibiotic-analgesic powder (Neopredef, Kalamazoo, MI) on incision sites. Gentamicin (5 mg/mL; Aspen Veterinary Resources, Ltd., Liberty, MO) in heparinized saline (Meitheal Pharmaceuticals, Chicago, IL) was flushed once daily (0.5 mL) during postoperative recovery for the first 3 days of recovery to prevent infection and ensure catheter patency. Heparinized saline was flushed daily for the remainder of the 5 day recovery period before beginning cocaine self-administration. During postoperative recovery, rats were housed in their home cage.

### Self-Administration

Rats were housed in operant chambers equipped with ad libitum food and water and allowed to self-administer cocaine from 10:00 to 16:00 daily. Rats initially acquired cocaine self-administration on a 6 h fixed ratio (FR1), LgA schedule of reinforcement, where active lever presses resulted in cocaine delivery (0.75 mg/kg; 2.5 mg/mL), illumination of a cue light above the lever for the duration of the cocaine infusion, and a 20 s time-out when lever presses did not result in cocaine delivery, indicated by illumination of house light. Responses on the inactive lever were recorded but had no consequence. Once self-administration behavior was acquired (≥40 injections in two consecutive sessions; maximum of ten sessions), rats were switched to the IntA schedule. During the 6 h IntA sessions, rats had repeated cycles of FR1 access to cocaine during 5 min periods followed by 25 min periods when levers were retracted for a total of 1 h of lever availability. Active lever presses resulted in cocaine delivery (0.375 mg/kg; 5 mg/mL) and illumination of a cue light above the lever for the duration of the cocaine infusion. There was no timeout period following each infusion to allow for a binge-like pattern of consumption. Inactive lever responses were recorded but had no consequence. This protocol has been shown to promote cue-induced cocaine seeking, DA uptake, and DAT sensitivity to cocaine (i.e., increased cocaine-induced inhibition of DA uptake) during abstinence.^1,80,95^

### Forced Abstinence and Cue-Induced Seeking

To determine if repeated treatment of unbiased and G-protein biased D3R agonists mitigates incubation of cocaine seeking, rats received daily treatment with saline, PRX (0.25 mg/kg), or SK609 (4 mg/kg) and were assessed for cue-induced cocaine seeking. After 7 IntA sessions, rats entered a forced abstinence period during which they resided in their home cage except for cue-induced seeking tests on AD1 and AD7. To measure cue-induced drug seeking, rats were presented with all self-administration cues during 1 h FR1 sessions but did not receive cocaine injections following an active lever press. Cocaine seeking was quantified by active lever presses during seeking tests, beginning at 10:00 on AD1 and AD7. Beginning on AD2, rats received daily ip injections at 09:30 for the remainder of abstinence, including 30 min before the AD7 seeking test and 30 min before euthanasia for Western blotting and FSCV on AD8.

### Statistical Analyses

Statistical tests were performed using Graph Pad Prism (Version 8, La Jolla, CA) or IMB SPSS Statistics (Version 24, Chicago, IL). Specific tests performed are included in the results section and figures. For all statistically significant comparisons, Dunnett’s posthoc tests were implemented to compare each treatment (PRX and SK609) to saline. For experiments that used an internal control rather than a separate control group (i.e., time course assays without cocaine in Figure 1), S̆ídák’s posthoc tests were implemented to compare treatment groups. One sample in the saline/SK609 group was removed from analysis of tDAT, pDAT, and pDAT/tDAT as it was identified as a statistical outlier using Grubbs’ test (Figure 2). For analysis of group differences for self-administration session parameters (i.e., lever presses and infusions per session), a mixed-effects model was used due to a missing value for one rat on 1 day because of a technical error in the data collection software. However, the rat did receive the appropriate amount of cocaine for the session and remains included (Figure 4).

## ABBREVIATIONS

DA: dopamine
DAT: dopamine transporter
D3R: dopamine D3 receptor
Thr53: Threonine 53
pDAT: phosphorylated dopamine transporter
ERK1/2: extracellular signal-regulating kinase 1/2
PKC: protein kinase C
NAc: nucleus accumbens
PRX: pramipexole
FSCV: fast-scan cyclic voltammetry
SH-SY5Y-D3R: human SH-SY5Y neuroblastoma cell line with stable expression of D3R
tDAT: total dopamine transporter
pDAT/tDAT: ratio of phosphorylated dopamine transporter to total dopamine transporter
CC: cocaine
SAL: saline
AD: abstinence day
LgA: long access
IntA: intermittent access
WB: Western blot
DMEM: Dulbecco’s Modified Eagle Medium
PBS: phosphate buffered saline
aCSF: artificial cerebrospinal fluid
i.p.: intraperitoneal
FR1: fixed ratio 1
PMSF: phenylmethylsulfonyl fluoride
PVDS: polyvinylidene fluoride
DREADDs: designer receptors exclusively activated by designer drugs
